# The Improvement of Bioethanol Production by Pentose-Fermenting Yeasts Isolated from Herbal Preparations, the Gut of Dung Beetles, and Marula Wine

**DOI:** 10.1155/2020/5670936

**Published:** 2020-07-13

**Authors:** Mahlatse Ellias Moremi, Elbert Lukas Jansen Van Rensburg, Daniël Coenrad La Grange

**Affiliations:** Department of Biochemistry, Microbiology and Biotechnology, University of Limpopo, Private Bag X1106, Sovenga 0727, South Africa

## Abstract

Efficient conversion of pentose sugars to ethanol is important for an economically viable lignocellulosic bioethanol process. Ten yeasts fermenting both D-xylose and L-arabinose were subjected to an adaptation process with L-arabinose as carbon source in a medium containing acetic acid. Four *Meyerozyma caribbica*-adapted strains were able to ferment L-arabinose to ethanol in the presence of 3 g/L acetic acid at 35°C. *Meyerozyma caribbica* Mu 2.2f fermented L-arabinose to produce 3.0 g/L ethanol compared to the parental strain with 1.0 g/L ethanol in the absence of acetic acid. The adapted *M. caribbica* Mu 2.2f strain produced 3.6 and 0.8 g/L ethanol on L-arabinose and D-xylose, respectively, in the presence of acetic acid while the parental strain failed to grow. In a bioreactor, the adapted *M. caribbica* Mu 2.2f strain produced 5.7 g/L ethanol in the presence of 3 g/L acetic acid with an ethanol yield and productivity of 0.338 g/g and 0.158 g/L/h, respectively, at a *K*_*L*_*a* value of 3.3 h^−1^. The adapted strain produced 26.7 g/L L-arabitol with a yield of 0.900 g/g at a *K*_*L*_*a* value of 4.9 h^−1^.

## 1. Introduction

Biofuels are increasingly becoming a renewable alternative to fossil fuels, and it is estimated that by 2035 approximately one-quarter of the world's energy will be generated from plant biomass [1]. First-generation bioethanol is produced from food crops, such as maize and sugarcane [2]. However, first-generation bioethanol raises concern about environmental impacts. The main disadvantage of first-generation bioethanol is the use of food crops for the production of fuel, which could lead to food shortages and rising food prices [3]. Second generation bioethanol is produced from lignocellulosic biomass, such as forest resources, agricultural residues, and municipal wastes. These biomass sources are abundant and cheap and do not compete with food resources, directly or indirectly [1, 4, 5]. Therefore, the conversion of abundant lignocellulosic biomass to liquid transportation fuel will help improve energy security globally [6].

Lignocellulose is composed of three polymeric components, namely, cellulose, hemicellulose, and lignin. Cellulose is an unbranched polysaccharide consisting of glucose units, while hemicellulose is a branched polysaccharide consisting of hexose (mainly D-mannose and D-galactose) and pentose (mainly D-xylose or L-arabinose) sugars residues, which often also has acetyl groups [7]. Hemicellulose and cellulose contribute up to 70% (dry weight) of plant biomass and are covalently linked to lignin [8]. Production of lignocellulose based ethanol involves pretreatment, hydrolysis, and fermentation. Pretreatment of lignocellulosic biomass is necessary to provide enzymes access to the polysaccharides (hemicellulose and cellulose) in plant biomass in order to ensure efficient saccharification [1]. Unfortunately, pretreatment often results in the release of toxic acidic compounds, like acetic acid, which negatively affect yeast during the fermentation step [3]. The hydrolysis step involves the release of simple sugars from the cellulose and hemicellulose components, which can then be converted to ethanol during the fermentation step [9].

D-xylose and L-arabinose released from hemicellulose generally constitute a significant fraction (nearly 16–19% and 3–15%, respectively) of lignocellulosic biomass. Therefore, their conversion to bioethanol is essential for an economically feasible production process [2, 10]. Fermentation of glucose to bioethanol by yeasts is well known, while the ability of microorganisms to convert D-xylose and L-arabinose to ethanol is often problematic. This is due to the lack of robust microorganisms that can ferment pentose sugars effectively in the presence of inhibitors, like acetic acid released during pretreatment at elevated temperatures [11, 12]. One approach to improve pentose-fermenting yeasts in the presence of inhibitors and high temperatures is adaptation or evolutionary engineering [13]. There is a need to develop yeast strains able to ferment a wide variety of sugars (pentoses and hexoses) in a highly inhibitory environment (with acetic acid often present) and high temperatures, while maintaining a high ethanol yield and production rate [14]. Therefore, this study aimed to improve yeasts isolated from the gut of dung beetles, herbal concoctions, marula wine, and banana wastes for their ability to ferment D-xylose and L-arabinose in the presence of acetic acid at elevated temperatures.

## 2. Materials and Methods

### 2.1. Yeasts

Three hundred and ninety yeasts, previously isolated from banana waste, the gut of dung beetles, herbal concoctions, and marula wine were streaked on D-xylose or L-arabinose agar plates (10 g/L D-xylose or L-arabinose, 15 g/L bacteriological agar, 6.7 g/L yeast nitrogen base (YNB), and 0.2 g/L chloramphenicol) and incubated at 30°C for five days. Yeast isolates were inoculated on freshly inoculated yeast malt agar (YM) slants (10 g/L glucose, 0.2 g/L chloramphenicol, 3 g/L malt extract, 3 g/L yeasts extract, 5 g/L peptone, and 15 g/L bacteriological agar) and incubated at 30°C for 24 hours before moving to 4°C for long term storage [15]. All pentose sugars in this study were autoclaved separately and mixed with the rest of the medium after autoclaving to prevent caramelization. The purity of yeast cultures was regularly checked by microscopic examination and colony morphology, throughout the study.

### 2.2. Selection of D-Xylose and L-Arabinose Fermenting Yeasts

All yeasts capable of growing on both D-xylose and L-arabinose agar plates were inoculated into test tubes with a Durham tube. These test tubes contained modified fermentation media as described by Silva et al. [16] consisting of 30 g/L sugar (D-xylose or L-arabinose), 5 g/L peptone, 3 g/L yeast extract, 2.3 g/L urea, 3 g/L KH_2_PO_4_, 1 g/L MgSO_4_, and 0.2 g/L chloramphenicol. The fermentation test tubes were incubated at 30°C for five days. Sugar fermentation was indicated by the presence of a gas bubble in the Durham tube. *Scheffersomyces stipitis* NRRL Y-7124 was used as a positive control for both D-xylose and L-arabinose fermentation. Experiments were performed in duplicate.

### 2.3. Yeast Identification Using ITS and D1/D2 Sequencing

All yeast isolates capable of fermenting D-xylose and L-arabinose were identified using DNA sequencing as described by Makhuvele et al. [17]. The ZR Fungal/Bacterial DNA MiniPrep^TM^ Kit (Zymo Research) was used for genomic DNA extraction, according to the instructions of the manufacturer. The ITS1 region of all selected yeasts was amplified using the PCR primers ITS-1 (5′-TCC GTA GGT GAA CCT GCG G-3′) and ITS-4 (5′-TCC TCC GCT TAT TGA TAT GC-3′) [15]. Amplification was done in 25 *µ*l reactions using the EconoTaq Plus Green Master Mix (Lucigen). The following PCR conditions were used: an initial denaturation at 95°C for 2 min followed by 35 cycles of denaturation at 95°C for 30 s, annealing at 50°C for 30 s, and extension at 72°C for 1 min. A final extension at 72°C for 10 min was followed by holding at 4°C. The D1/D2 domain of the 26S rDNA region was also amplified for all yeast isolates using primers NL1 (5′-GCA TAT CAA TAA GCG GAG GAA AAG-3′) and NL4 (5′-GGT CCG TGT TTC AAG ACG G-3′) as described above. The DNA sequencing was done with ABI V3.1 BigDye according to the manufacturer's instructions on the ABI 3500 XL Instrument by Inqaba Biotechnical Industries (Pty) Ltd (South Africa).

Sequence data were cleaned using Chromas software (Technelysium Pty, South Brisbane, Australia) followed by BioEdit [18] to produce a consensus sequence from the forward and reverse reads. Yeast isolates were identified by comparing the obtained sequences with that of the National Center for Biotechnology Information (NCBI) database (http://www.ncbi.nlm.nih.gov/BLAST/) using the Basic Local Alignment Search Tool (BLAST). The sequences obtained were deposited in GenBank.

### 2.4. Ethanol Production by Pentose-Fermenting Yeasts

Precultures were prepared in 250 ml Erlenmeyer flasks containing 25 ml of modified fermentation media as described earlier with D-xylose or L-arabinose as carbon source. The flasks were inoculated with yeasts able to ferment both D-xylose and L-arabinose and incubated at 30°C with shaking at 200 rpm for 48 hours. The cultures were used to inoculate 250 ml Erlenmeyer flasks containing 100 ml of the same media to a starting optical density (OD_600nm_) of 0.1. The flasks were then incubated as above for 96 hours. Two-millilitre samples were withdrawn at 24, 48, 72, and 96 hours to determine the ethanol concentration using gas chromatography (GC). Duplicate cultures were prepared for each yeast.

### 2.5. Acetic Acid and Thermotolerance of Pentose-Fermenting Yeasts

Pentose-fermenting yeasts able to ferment both pentose sugars were grown on slants containing 6.7 g/L YNB and 20 g/L of L-arabinose and incubated at different temperatures (35, 37, and 40°C) to determine the maximum growth temperature. Different concentrations of acetic acid (1, 2, and 3 g/L) were added in the same media (used for temperature evaluation) to determine the ability of the yeasts to grow in the presence of acetic acid during incubation at 30°C. Yeasts with acetic acid tolerance and thermotolerance were selected for evolutionary engineering. These experiments were done in duplicate.

### 2.6. Evolutionary Engineering of Yeasts on L-Arabinose

Yeast strains able to ferment both pentose sugars in the presence of 3 g/L acetic acid were inoculated onto agar plates containing 6.7 g/L YNB supplemented with 30 g/L L-arabinose and 3 g/L acetic acid and incubated at 35°C for 24 hours at pH 5.0. Colonies were restreaked onto the same media and incubated at 35°C for 24 hours, and the process was repeated 50 times. The process was then repeated 50 times with incubation at 37°C followed by 40°C [19].

### 2.7. Screening of Adapted Yeasts for Ethanol Production

The best-adapted yeast strains (ability to ferment in the presence of acetic acid at elevated temperatures) were screened for ethanol production at different temperatures (35, 37, and 40°C) in 250 ml Erlenmeyer flasks containing modified fermentation medium (30 g/L L-arabinose, 3 g/L yeast extract, 5 g/L peptone, 2.3 g/L urea, 1 g/L MgSO_4_, 3 g/L KH_4_PO_2_, and 0.2 g/L chloramphenicol) with 3 g/L acetic acid. Each flask contained L-arabinose as carbon source, and sampling was done every 24 hours for 5 days.

The best-adapted yeast strain was selected based on high ethanol production along with acetic acid tolerance and thermotolerance. This yeast was subsequently compared with the parental strain in terms of fermentation ability on different pentose sugars in the presence and absence of acetic acid. The following pentose sugar concentrations were used: 50 g/L D-xylose, 40 g/L L-arabinose, or a mixture of 50 g/L D-xylose and 40 g/L L-arabinose with or without the addition of 3 g/L acetic acid. Experiments were conducted in triplicate with sampling every 24 hours for 5 days. High-performance liquid chromatography (HPLC) was used to determine D-xylose, L-arabinose, L-arabitol, and D-xylitol concentrations, with gas chromatography (GC) used for ethanol determination.

### 2.8. Fermentation Studies

The best-adapted yeast strain was further evaluated for ethanol production in a BioFlo New Brunswick Bioreactor using a three-litre fermenting vessel containing one litre of media. The same fermenting media as indicated earlier was used with L-arabinose as carbon source. Fermentation in the bioreactor was conducted at a fixed pH of 5.0 by adding 3M HCl to prevent an increase in media pH at 35°C. Aeration and agitation were used to maintain different fixed volumetric oxygen transfer coefficient (*K*_*L*_*a*) values (2.3, 3.3, and 4.9 h^−1^). The experiments were conducted in triplicate for a period of 120 hours. Sampling was done regularly to determine biomass, ethanol, L-arabinose, and L-arabitol concentrations in the bioreactor. Biomass determinations were done using dry weight in grams, whereas sugar and ethanol concentrations were determined using HPLC and GC, respectively.

### 2.9. Determination of Volumetric Oxygen Transfer Coefficient (*K*_*L*_*a*)

Various aeration rates and agitation speeds were used to determine the effect of oxygen on ethanol production by the selected adapted yeast strain. The dynamic gassing-out method was applied to determine the different *K*_*L*_*a* (2.3, 3.3, and 4.9 h^−1^) values. In this method, the oxygen concentration in the uninoculated medium was reduced to zero by gassing in nitrogen gas. The deoxygenated medium was reaerated and agitated at a fixed agitation speed and aeration rate using a calibrated polarographic oxygen sensor to measure dissolved oxygen in the medium. The polarographic oxygen sensor was previously calibrated at atmospheric pressure according to the instructions of the manufacturer. The concentration of dissolved oxygen in the medium was monitored using the following equation:

(1) $$\begin{array}{c} \frac{\text{d}CL}{\text{d}t}=K_{L}a\left(C*L-C_{L}\right). \end{array}$$

The *K*_*L*_*a* values were calculated using ln(*C∗L* − *C*_*L*_) versus time, where *C*_*L*_ is the concentration of dissolved oxygen in the fermentation broth and *C∗L* is the saturated dissolved oxygen concentration in the fermentation medium [20]. The *K*_*L*_*a* values tested during fermentation of L-arabinose were 2.3 h^−1^, 3.3 h^−1^, and 4.9 h^−1^ with air that was introduced into the bioreactor at 0.1 vvm for all *K*_*L*_*a* values with the agitation speed at 100 rpm for 2.3 h^−1^, 150 rpm for 3.3 h^−1^, and 200 rpm for 4.9 h^−1^. The range of different *K*_*L*_*a* values used was the same as described by Silva et al. [16] where D-xylose was used as carbon source.

## 3. Analytical Methods

### 3.1. GC Analysis

The ethanol content was determined with a GC-2010 Plus Shimadzu Gas Chromatograph. A ZB-WAX plus column was used at a starting temperature of 40°C and raised to 140°C after sample injection at a rate of 20°C/min. It was then raised to 200°C at a rate of 50°C/min and kept at this temperature for 2 min. Nitrogen was used as carrier gas at a flow rate of 17.6 mL/min and at a pressure of 100 kPa. The temperature of the detector was set at 255°C. For each sample, a volume of 1 *µ*L was automatically injected onto the GC column using a split syringe AOC-20i + *s*. The ethanol in the samples was measured by comparing it with known ethanol standards [4].

### 3.2. HPLC Analysis

A Shimadzu prominence 20 (Tokyo, Japan) HPLC instrument equipped with a RID 10A Refractive Index detector was used to quantify D-xylose, L-arabinose, L-arabitol, and D-xylitol. A Rezex RHM-Monosaccharide *H* + (300 mm × 7 mm) column was used, and deionized water was used as the mobile phase. The temperature was set at 85°C, and the flow rate was 0.6 ml/min. A sample volume of 20 *µ*l was injected onto the column using a SIL-20A autosampler. LC Solution Operation analysis was used to process sample data. Known standards of D-xylose, L-arabinose, L-arabitol, and D-xylitol were used to calculate the concentration unknowns [17].

### 3.3. Calculations of Fermentation Parameters

Fermentation parameters, L-arabitol, cell biomass, ethanol and D-xylitol yield, ethanol productivity D-xylose, and L-arabinose consumption were determined as described below. L-arabitol (g/g), ethanol (g/g), and D-xylitol (g/g) yields were calculated as described by Cadete et al. [21], which correlated to the products generated (Δ*P*_arabitol_, Δ*P*_ethanol_, Δ*P*_xylitol_) from the substrates (Δ*S*_L−arabinose_, Δ*S*_D−xylose_) consumed. The ethanol productivity was calculated from the ratio between maximum ethanol concentration and fermentation time (h) at which the highest ethanol was observed. Cell concentrations were determined by correlating the optical density (OD) measurements spectrophotometrically at 600 nm with a standard curve of dry weight against optical density previously constructed. The biomass yield was determined by the ratio between cell concentration (g/L) and substrate utilized (g/L).

## 4. Results

In this study, a total of 390 yeasts, previously isolated from banana waste, the gut of dung beetles, marula wine, and herbal concoctions, were evaluated for the ability to grow and ferment both D-xylose and L-arabinose. Twenty-seven yeasts were able to grow on plates containing either D-xylose or L-arabinose, and 13 yeasts were able to ferment both sugars. Yeasts with preferred characteristics (growth at elevated temperatures in the presence of acetic acid) were selected for adaptation. The adapted yeasts were evaluated to select the best ethanol producing strain. The best-adapted yeast strain was evaluated in a bioreactor to determine the optimum aeration rate for ethanol production.

### 4.1. Identification of Selected Yeasts

Thirteen yeast isolates capable of D-xylose and L-arabinose fermentation were identified using ITS-5.8S and D1/D2 domain sequencing (Table 1). Most of the yeast isolates identified belong to *Meyerozyma caribbica* (D28L3, D14W2, D28L4, D14YE6, D14YE1, D14YE2, D4WPO1, and Mu 2.2f) followed by *Cryptococcus terrestris* (C11Y, C12Y, CW1, and CW2) and *Candida tropicalis* (Kp34ey).

### 4.2. Ethanol Production, Acetic Acid Tolerance, and Maximum Growth Temperature

Thirteen yeasts able to ferment both D-xylose and L-arabinose were evaluated for ethanol production. Maximum ethanol produced after a certain fermentation time is indicated in Table 2. After incubating for 72 hours, *Meyerozyma caribbica* D14YE6 produced 3.9 g/L ethanol from L-arabinose, followed by *M. caribbica* D14W2 (1.9 g/L) and *M. caribbica* Mu 2.2f (0.7 g/L). As expected, the control strain *S. stipitis* NRRL Y-7124 produced the most ethanol (4.5 g/L) from D-xylose in 24 hours, followed by *M. caribbica* D14W2 and *C. tropicalis* Kp42ey with 1.2 g/L and 1.0 g/L of ethanol in 48 hours, respectively. The other yeasts produced less than 1 g/L of ethanol from D-xylose.

The thirteen yeast isolates were tested for the ability to grow at elevated temperatures and in the presence of acetic acid with L-arabinose as carbon source (Table 3). D-xylose was not used further as carbon source, because of the low levels of ethanol produced by the yeast isolates compared to *S. stipitis* NRRL Y-7124. All yeasts belonging to *Meyerozyma caribbica* (D28L3, D14W2, D28L4, D14YE6, Mu 2.2f, D14YE1, D14YE2, and D4WPO1) were able to grow in the presence of 3 g/l acetic acid on agar slants with growth observed after two to four days of incubation at 30°C. The yeast strains *C. tropicalis* Kp42ey and *S. stipitis* NRRL Y-7124 were able to grow in the presence of 1 g/l acetic acid, while all the strains of *C. terrestris* (C12Y, CW1, CW2, and C11Y) failed to grow in the presence of acetic acid. *Meyerozyma caribbica* D28L3, D14W2, D28L4, D14YE6, and Mu 2.2f were able to grow at a maximum temperature of 40°C, with *M. caribbica* D14YE1 and D14YE2 growing at 37°C. *Candida tropicalis* Kp42ey and *S. stipitis* NRRL Y-7124 were able to grow at 35°C, while all four strains belonging to *C. terrestris* (C12Y, CW1, CW2, and C11Y) grew at a maximum of 30°C along with *M. caribbica* D4WPO1.

### 4.3. Yeast Adaptation

Yeast strains with the ability to grow in the presence of acetic acid and at temperatures above 30°C were adapted on YNB agar plates containing L-arabinose. Ten yeast strains (*C. tropicalis* Kp42ey; *M. caribbica* D28L3, D14W2, D28L4, D14YE6, Mu 2.2f, D14YE1, D14YE2, D4WPO1; and *S. stipitis* NRRL Y-7124) were selected for adaptation. For the initial step of adaptation, yeasts were grown on agar plates containing 3 g/L acetic acid at 35°C. Yeast strains that grew during the first stage of adaptation were adapted further to 3 g/L acetic acid at 37°C and subsequently on agar plates with 3 g/L acetic acid at 40°C. *Candida tropicalis* Kp42ey and *S. stipitis* NRRL Y-7124 failed to grow on agar plates containing 3 g/L acetic acid when incubated at 35°C, while *M. caribbica* D14YE2, D4WPO1, D14W2, and D28L4 failed to grow on agar plates with 3 g/L acetic acid at 40°C. Only *M. caribbica* D28L3, D14YE1, D14YE6, and Mu 2.2f were able to adapt to the most stringent conditions.

### 4.4. Shake Flask Fermentation Studies on Adapted Yeast Strains

The four adapted *M. caribbica* strains (D28L3, D14YE1, D14YE6, and Mu 2.2f) were screened for L-arabinose (30 g/L) fermentation at 35 and 37°C. Although these yeasts were capable of growth on agar plates at 40°C, none of them were able to grow in liquid medium at 40°C. At 35°C, L-arabinose containing liquid medium with 3 g/L acetic acid, *M. caribbica* Mu 2.2f, and *M. caribbica* D14YE1 produced 4.3 g/L and 1.2 g/L ethanol in 24 hours, respectively (Table 4). This is a significant improvement when compared to 0.7 g/L and 0.5 g/L ethanol, respectively, produced by the parental strains without the addition of acetic acid to the medium (Table 2). The ethanol yield for the adapted *M. caribbica* strains on L-arabinose varied between 0.030 and 0.160 g/g with *M. caribbica* Mu 2.2f having the highest ethanol yield (Table 4). It was also noted that *M. caribbica* Mu 2.2f had the highest ethanol productivity of 0.180 g/L/h followed by *M. caribbica* D14YE1 with an ethanol productivity of 0.050 g/L/h. Both these ethanol productivity values were calculated after 24 hours. The L-arabitol yield was between 0.783 and 0.764 g/g with *M. caribbica* D14YE6 producing 22 g/L L-arabitol followed by *M. caribbica* D14YE1 with 21.6 g/L L-arabitol after 72 and 96 hours, respectively.

Ethanol production, yield, and productivity of the adapted yeasts grown on fermentation medium containing acetic acid at 37°C are presented in Table 5. All four yeast strains were able to grow with *M. caribbica* Mu 2.2f producing 1.7 g/L ethanol in 48 hours and *M. caribbica* D14YE6 producing 0.8 g/L ethanol in 24 hours. Ethanol production in the adapted strain of *M. caribbica* Mu 2.2f increased from 0.7 g/L in the parental strain (without acetic acid) to 1.7 g/L in the adapted strain (with acetic acid). The adapted strain of *M. caribbica* Mu 2.2f produced ethanol at a yield and productivity of 0.221 g/g and 0.047 g/L/h, respectively, in L-arabinose with acetic acid. The yeast *M. caribbica* D28L3 consumed most of the L-arabinose (24.7 g/L, data not shown) and converted it mostly to L-arabitol (19.2 g/l) rather than ethanol (0.6 g/L). This strain had an L-arabitol yield of 0.792 g/g compared to *M. caribbica* D14YE6 (second best L-arabitol producer) producing 6.6 g/L L-arabitol with a yield of 0.299 g/g. It was decided that *M. caribbica* Mu 2.2f should be investigated further, since it produced the most ethanol in the presence of acetic acid at 35 and 37°C.

### 4.5. Comparison of Adapted and Parental Strains of *M. caribbica* Mu 2.2f

The adapted and parental strains of *M. caribbica* Mu 2.2f were evaluated for their ability to ferment pentose sugars in the presence and absence of 3 g/L acetic acid (Table 6). Both strains were able to grow and ferment in medium containing 50 g/L D-xylose or 40 g/L L-arabinose or a 90 g/L pentose mixture consisting of 50 g/L D-xylose and 40 g/L L-arabinose, without acetic acid in the medium. The adapted strain produced 1.9 g/L ethanol compared to 1.8 g/L ethanol for the parental strain from the pentose mix after 36 hours of fermentation with ethanol yields of 0.059 and 0.052 g/g, respectively. The ethanol productivity of the adapted strain was slightly higher (0.053 g/L/h) compared to 0.04 g/L/h for the parental strain. The parental strain produced more D-xylitol (6.5 g/L) from the pentose mixture than the adapted strain (4.6 g/L), while the adapted strain produced more L-arabitol (4.7 g/L) than the parental strain (2.2 g/L). The L-arabitol and D-xylitol yields of the parental strain were 0.381 g/g and 0.150 g/g, respectively, whereas the adapted strain had yields of 0.347 g/g and 0.084 g/g for L-arabitol and D-xylitol, respectively. It consumed less of the pentose sugars compared to the parental strain (data not shown).

The adapted strain produced a maximum of 3.0 g/L ethanol from L-arabinose compared to the maximum of 1.0 g/L ethanol for the parental strain (Table 6). The adapted strain also produced a higher ethanol yield and productivity of 0.148 g/g and 0.062 g/L/h, respectively, compared to the parental strain (0.076 g/g and 0.043 g/L/h, respectively) with L-arabinose as carbon source. The adapted strain produced more L-arabitol (16.8 g/L) with a higher yield (0.494 g/g) than the parental strain with 7.4 g/L L-arabitol at a yield of 0.325 g/g.

The adapted strain produced 1.7 g/L ethanol with a yield of 0.042 g/g from D-xylose compared to 1.5 g/L ethanol at a yield of 0.044 g/g for the parental strain. The maximum ethanol productivity for the adapted strain was 0.071 g/L/h compared to 0.063 g/L/h for the parental strain. However, the parental strain produced a higher D-xylitol concentration of 8.5 g/L and a D-xylitol yield of 0.2 g/g compared to 2.8 g/L ethanol produced with a yield of 0.06 g/g for the adapted strain.

The parental strain could not grow on any of the pentose sugars in the presence of acetic acid. The adapted strain could not also grow on a combination of D-xylose and L-arabinose in the presence of acetic acid. However, the adapted strain fermented 50 g/L D-xylose and 40 g/L L-arabinose separately in the presence of 3 g/L acetic acid. The adapted strain produced more ethanol from L-arabinose (3.6 g/L) than from D-xylose (0.8 g/L) after 36 hours of fermentation (Table 6). The ethanol yield and productivity of the adapted strain were also higher on L-arabinose with 0.181 g/g and 0.100 g/L/h, respectively, than on D-xylose with an ethanol yield of 0.04 g/g and a productivity of 0.02 g/L/h. It was also noted that the adapted strain produced 3.8 g/L D-xylitol with a yield of 0.172 g/g on D-xylose and 20.8 g/L L-arabitol with a yield of 0.657 g/g on L-arabinose. Bioreactor fermentation studies were conducted to determine the effect of different aeration strategies of *M. caribbica* Mu 2.2f with 30 g/L L-arabinose as carbon source in the presence of acetic acid at 35°C, since these conditions resulted in a higher ethanol production, when acetic acid was present.

### 4.6. Effect of Aeration on L-Arabinose Fermentation

**Figure 1:**
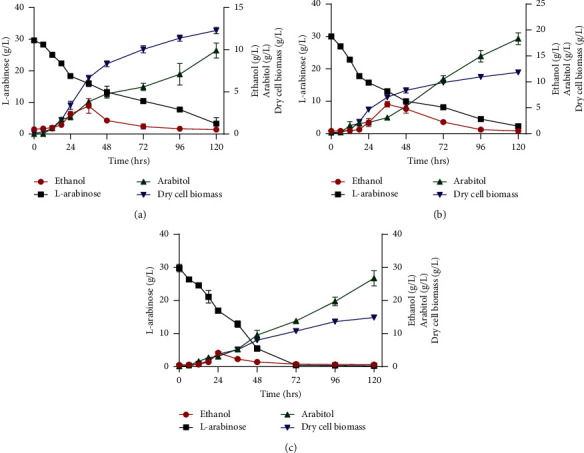
Fermentation of L-arabinose by the adapted strain of *M. caribbica* Mu 2.2f at K_L_a values of 2.3 h^−1^ (a), 3.3 h^−1^ (b), and 4.9 h^−1^ (c) in the presence of 30 g/L L-arabinose at 35°C with the addition of 3 g/L acetic acid.

The adapted strain of *M. caribbica* Mu 2.2f was evaluated at different volumetric oxygen transfer coefficients (*K*_*L*_*a* values) in a bioreactor in order to determine the ideal aeration strategy for maximum ethanol production and L-arabinose consumption. The temperature of the bioreactor was controlled at 35°C and the pH kept at 5.0 with 30 g/L of L-arabinose as carbon source. The adapted strain produced the highest ethanol concentration (5.7 g/L) at a *K*_*L*_*a* value of 3.3 h^−1^ after 36 hours compared to 4.2 g/L for *K*_*L*_*a* 4.9 h^−1^ and 3.3 g/L for *K*_*L*_*a* 2.3 h−^1^ (Figure 1, Table 7). The ethanol yield was the highest at K_L_a 3.3 _h_^−^1 (0.338 g/g). The ethanol productivity at *K*_*L*_*a* 4.9 h^−1^ was 0.175 g/L/h, compared to 0.158 g/L/h for *K*_*L*_*a* 3.3 h^−1^ and 0.106 g/L for 2.3 h^−1^. The maximum ethanol produced at *K*_*L*_*a* 4.9 h^−1^ was obtained after 24 hours, which contributed to the higher ethanol productivity, while at *K*_*L*_*a* 2.3 h^−1^ and 3.3 h^−1^ the maximum ethanol production was observed after 36 hours.

However, the adapted strain produced 26.7 g/L L-arabitol at a *K*_*L*_*a* value of 4.9 h^−1^, 18.2 g/L at *K*_*L*_*a* 3.3, and 9.9 g/L at *K*_*L*_*a* 2.3 h^−1^. Similarly, the L-arabitol yield was 0.9 g/g at *K*_*L*_*a* 4.9 h^−1^, compared to 0.66 g/g for 3.3 h^−1^ and 0.37 g/g for *K*_*L*_*a* 2.3 h^−1^. Furthermore, L-arabinose present in the fermentation medium was fully consumed at a *K*_*L*_*a* value of 4.9 h^−1^ (Figure 1(c)) after 120 hours of fermentation.

## 5. Discussion

The excessive use of nonrenewable fossil fuel as an energy source worldwide has resulted in an increased release of greenhouse gases into the atmosphere that is leading to global warming [22]. Biofuel (mainly ethanol) has been regarded as a viable alternative clean and renewable energy source to fossil fuels. Efficient second generation production of bioethanol from plant materials requires efficient conversion of all sugars including hexose and pentose sugars present in plant biomass. The hexose sugars (glucose, galactose, and mannose) in hemicellulose are efficiently converted to ethanol by the traditional fermenting yeast, *S. cerevisiae*; however, pentose sugars (D-xylose and L-arabinose) are not naturally fermented by *S. cerevisiae*. It is crucial to convert both hexose and pentose sugars to bioethanol for the process to be economically feasible [12].

Yeasts previously isolated from the gut of dung beetles, herbal concoctions, banana waste, and marula wine were screened for the ability to ferment both D-xylose and L-arabinose. Seven of the yeast isolates used in this study were obtained from marula wine. Yeasts associated with marula wine are typically good fermenters. Molelekoa et al. [23] isolated non-*Saccharomyces* yeast from marula fruit and found *Pichia kudriavzevii,* a yeast known for its pentose-fermenting ability. This yeast was investigated by several authors for its ability to produce ethanol from D-xylose [24–27].

Four yeast isolates (CW1, CW2, C12Y, and C11Y) used here were purified from herbal concoctions. There is no information available on the screening of yeasts associated with herbal concoctions in terms of pentose fermentation. Only one yeast isolate from the gut of dung beetles and one from banana waste, respectively, were able to ferment both pentose sugars. These sources are known to be associated with pentose-fermenting organisms. Suh et al. [28] isolated xylose assimilating and fermenting yeasts (*C. shehatae*, *C. ergatensis*, *S. stipitis*, *and S. segobiensis*) from passalid beetles. Makhuvele et al. [17] isolated 6 xylose assimilating yeasts belonging to *Candida tropicalis* from the dung of dassie, kudu, rhino, and wildebeest. Santa-Maria et al. [29] determined the pentose concentrations in different parts of banana waste, pseudostem (5–11% D-xylose and 2–3% L-arabinose), leaves (7–11% D-xylose and 3–4% L-arabinose), and rachis (8–11% D-xylose and 3-4% L-arabinose). Brooks [30] isolated 8 yeasts from banana peels for the production of ethanol, and all isolates failed to ferment D-xylose and L-arabinose.

Almost 400 yeast isolates were screened for their ability to ferment both D-xylose and L-arabinose in this study. Only 13 isolates were able to produce gas in Durham tubes (data not shown) on both pentose sugars. Araújo et al. [31] screened xylose-fermenting ability among 205 yeast isolates obtained from fruit pulp and plants of Cerrado. They found that only 3 isolates were able to ferment D-xylose in test tubes. One of the 3 isolates was identified as *Meyerozyma guilliermondii,* a close relative of *M. caribbica*. Martini et al. [32] isolated 350 yeasts from sugarcane, and only one isolate fermented both D-xylose and L-arabinose in test tubes. This yeast isolate was also identified as *M. guilliermondii*. Species of *Meyerozyma* isolated from marula wine dominated, as 8 isolates were identified as *M. caribbica* and four as *Cryptococcus terrestris*, with only one strain of *C. tropicalis* isolated (Table 1). Martini et al. [32] isolated yeasts from sugarcane juice, and the best pentose-fermenting yeast, *M. guilliermondii,* fermented both D-xylose and L-arabinose. There is not much information available on the fermentation of pentoses by *M. caribbica.* Studies mostly indicate that low or no ethanol was detected [33–35]. *Meyerozyma caribbica* is regarded as a safe and harmless yeast as it is used in Mexico for the production of tequila [36].

Four basidiomycetous yeasts, isolated from herbal concoctions, were identified as *C. terrestris* (Table 1). Yeasts belonging to *Cryptococcus* are not known for their fermenting abilities. Rao et al. [37] isolated xylose-fermenting yeasts from the bark of trees and found that basidiomycetous species such as *Rhodotorula* and *Cryptococcus* have the ability to ferment D-xylose and produce ethanol.

In this study, a number of *M. caribbica* strains isolated were able to ferment both D-xylose and L-arabinose (Table 2). The most ethanol measured during fermentation of D-xylose was 1.2 g/L (after 48 hours) by *M. caribbica* D14W2 and 3.9 g/L (after 72 hours) on L-arabinose by *M. caribbica* D14YE6 (Table 2). This is the first report of this yeast associated with marula wine with the ability to ferment both D-xylose and L-arabinose. In a study by Kurtzman and Dien [38], the authors found L-arabinose fermentation to be slow for wild-type yeast. *Candida arabinofermentans* YB-1984 produced 1.9 g/L ethanol from L-arabinose after 14 days; however, in this study the maximum ethanol concentration by a *M. caribbica* strain was obtained after 72 hours. A study by Dien et al. [33] on L-arabinose fermentation showed that *Ambrosiozyma monospora* NRRL Y-148 produced a maximum ethanol concentration of 4.1 g/L from L-arabinose after 12 days. Sukpipat et al. [35] used *M. caribbica* 5XY2 that was isolated from an alcohol starter fermentation to ferment D-xylose and L-arabinose. However, the yeast produced less than 0.6 g/L of ethanol from both pentose sugars after 120 hours.

Inhibitory compounds such as furans, weak acids, and phenolic compounds are normally produced during pretreatment of lignocellulosic biomass and have a negative effect on microorganisms involved in the fermentation of lignocellulose. Acetic acid is the inhibitor mostly studied due to its occurrence and severity of inhibition on the fermentation process. Acetic acid diffuses across the cytoplasmic membrane into the cell, and the dissociation of acetic acid that occurs inside the cytosol leads to a change in the intracellular pH. The decrease in the cytosolic pH may result in cell death [39–41]. All eight strains of *M. caribbica* were able to grow on plates with the addition of 3 g/L acetic acid (Table 3). The study conducted by Perna et al. [41] showed that *M. guilliermondii* CCT7783 grew on media containing L-arabinose and D-xylose in the presence of 10 g/L acetic acid and suggested that the species is capable of utilizing acetic acid as a carbon source. Charoensopharat et al. [26] investigated the effect of acetic acid (4, 6, 8, and 10 g/L) incorporated in YM agar plates on yeasts isolated from *Jerusalem artichoke*. The authors found that *M. caribbica* was among the yeast isolates able to grow on plates containing up to 4 g/L acetic acid.

The application of thermotolerant pentose-fermenting yeasts for bioethanol production possesses advantages over low-temperature ethanol fermentation. These include a higher hydrolysis rate for enzymes, ethanol yield, lower contamination risk, and lower cooling costs [27]. In this study, five strains of *M. caribbica* (D14W2, D14YE6, D28L3, D28L3, and Mu 2.2f) grew at 40°C on agar plates using L-arabinose as carbon source, and these strains were included in the adaptation process (Table 4). Sukpipat et al. [35] reported that a strain of *M. caribbica* 5XY2, isolated from an alcohol fermentation starter in Thailand, grew at 40°C. Charoensopharat et al. [26] also investigated the effect of temperature on yeast from *Jerusalem artichoke* and isolated an *M. caribbica* strain that could grow at 40°C on YM agar plates. Similarly, Kurtzman et al. [42] isolated a strain of *M. caribbica* that can grow up to a maximum temperature of 42°C on agar slants.

Evolutionary engineering or adaptation is used to improve certain traits of microorganisms, such as inhibitor tolerance, temperature sensitivity, or the production of bioethanol from lignocellulosic substrates [14, 43]. In this study, ten yeast strains (8 strains of *M. caribbica*, *C. tropicalis* Kp42ey, and *S. stipitis* NRRL Y-7124) were subjected to evolutionary engineering. Only four yeasts, all belonging to *M. caribbica* (D28L3, D14YE1, D14YE6, and Mu 2.2f), were capable of adapting up to 40°C in the presence of 3 g/l acetic acid. Strangely, no growth was observed when these yeasts were incubated in shake flasks at 40°C. It is known that oxygen solubility decreases with an increase in temperature in liquids (44). Therefore, it is possible that at 40°C in shake flasks, sufficient oxygen was not available to these yeasts to produce biomass. Similar results were reported by Abdel-Banat et al. [44], where *Kluyveromyces marxianus* DMKU3-1042 grew on YPD plates at 48°C but failed to grow at the same temperature when inoculated in flasks containing liquid medium. This study is the first report on the adaptation of *M. caribbica* on acetic acid and elevated temperature with L-arabinose as a carbon source.

The adapted *M. caribbica* Mu 2.2f strain was the best ethanol producing strain. This yeast was capable of producing 4.3 g/L ethanol after 24 hours during growth on L-arabinose in the presence of 3 g/L acetic acid at 35°C (Table 4). Ethanol yield (0.16 g/g) and productivity (0.180 g/L/h) were significantly higher than those of the other adapted *M. caribbica* strains. Ethanol production decreased to 1.7 g/L with a yield and productivity of 0.221 g/g and 0.047 g/L/h, respectively, when incubated at 37°C (Table 5). Similar results were obtained for *P. kudriavzevii* CM4.2 with more ethanol produced at 37°C than at 40°C, but using glucose as a carbon source [26]. Watanabe et al. [45] evaluated the effect of temperature on yeasts isolated from soil. *Candida* sp. NY7122 from this study produced 1.92 and 0.75 g/L ethanol with a yield of 0.11 and 0.04 g/g at 30 and 37°C from D-xylose, respectively.

All the adapted *M. caribbica* strains produced a significant amount of L-arabitol (20–22.2 g/L) during growth on L-arabinose, with *M. caribbica* D28L3 producing the most (22.2 g/L) with a yield of 0.783 g/g (Table 4). Kordowska-Wiater et al. [46] isolated L-arabitol producing yeasts from raspberry. One of their isolates, *C. parapsilosis* 27RL-4, produced 10.72 g/L L-arabitol with a yield of 0.53 g/g. Kordowska-Wiater et al. [47] reported that *S. shehatae* 20BM-3 from rotten wood produced 7.97 g/L L-arabitol with a yield of 0.36 g/g. Dien et al. [33] reported that *C. succiphila* Y-1998 and *C. auringiensis* Y-11848 produced 81 and 73 g/L L-arabitol from L-arabinose with yields of 1.01 g/g and 0.91 g/g, respectively.

The adapted strain of *M. caribbica* Mu 2.2f produced 3.6 g/L and 0.8 g/L ethanol from L-arabinose and D-xylose, respectively, with 3 g/L acetic acid in the medium at 35°C (Table 6). The parental strain was unable to ferment L-arabinose under these conditions. Similar results were obtained by Nigam [48], who adapted *S. stipitis* NRRL Y-7124 on hardwood hemicellulose acid hydrolysate. In this case, the adapted strain produced 8.3 g/L ethanol in the presence of 5 g/L acetic acid with the parental strain failing to produce ethanol. In the absence of acetic acid, the adapted strain *M. caribbica* Mu 2.2f produced 3-fold more ethanol from L-arabinose when compared to the parental strain. Similar results were obtained by various authors when *S. stipitis* was adapted on D-xylose, with the adapted yeast always producing more ethanol than the parental strain [19, 49]. The ethanol produced from D-xylose was similar for the adapted and the parental strain, since *M. caribbica* Mu 2.2f was only adapted on L-arabinose.

Bioreactor studies were used to control parameters such as agitation speed, aeration rate, pH, and temperature, which has an impact on the production of fermentation products (ethanol, L-arabitol, cell biomass, D-xylitol, etc.) [20, 50]. In order to determine the ideal aeration for optimal ethanol production during batch fermentation by the adapted strain of *M. caribbica* Mu 2.2f, different oxygen volumetric transfer coefficient (*K*_*L*_*a*) values (2.3 h^−1^, 3.3 h^−1^, 4.9 h^−1^) were used in a bioreactor (Figure 1 and Table 7). The maximum ethanol concentration by the adapted *M. caribbica* Mu 2.2f strain was observed at *K*_*L*_*a* 3.3 h^−1^ (5.7 g/L). Most studies investigating the effect of *K*_*L*_*a* on ethanol production were done on D-xylose as carbon source using yeasts such as *S. stipitis*, *S. hagerdaliae*, and *S. shehatae* [16, 20, 50, 51]. No studies were found reporting the optimization of aeration on L-arabinose fermentation. Bellido et al. [20] obtained the highest ethanol concentration, yield, and productivity of 22.3 g/L, 0.40 g/g, and 0.30 g/L/h, respectively, on D-xylose at a *K*_*L*_*a* of 3.3 h^−1^ for *S. stipitis* DSM 3651 after 72 hours in the absence of acetic acid. The adapted *M. caribbica* Mu 2.2f consumed all the L-arabinose at *K*_*L*_*a* 4.9 h^−1^ and produced the most biomass at this *K*_*L*_*a* (Figure 1(c)). Application of a high *K*_*L*_*a* value results in high sugar consumption but did not improve ethanol production [52].

L-arabitol is a five-carbon sugar alcohol often used as a natural sweetener in food and the pharmaceutical industry [53]. In this study, the adapted *M. caribbica* Mu 2.2f strain produced more L-arabitol (0.900 g/g) from L-arabinose than ethanol (0.338 g/g). Sukpipat et al. [35] investigated L-arabitol producing yeasts isolated from a alcohol fermentation starter in Thailand and noted that *M. caribbica* 5XY2 produced 30.3 g/L L-arabitol with a yield of 0.61 g/g from 50 g/L L-arabinose. Kumdam et al. [54] examined the production of L-arabitol from several lignocellulosic biomass sugars (sucrose, glucose, L-arabinose, fructose, and glycerol) using *Debaryomyces nepalensis* NCYC 3413 and reported significantly less ethanol (2.43 g/L) than L-arabitol (22.7 g/L) on L-arabinose with ethanol and L-arabitol yields of 0.03 and 0.26 g/g, respectively. Saha and Bothast [55] reported that strains of *Candida entomaea* NRRL Y-7785 and *S. guilliermondii* NRRL Y-2075 produced 33.0 and 31.5 g/L L-arabitol, respectively, from 50 g/L L-arabinose at 34°C with an L-arabitol yield of 0.66 and 0.63 g/g, respectively. Sundaramoorthy and Gummadi [56] isolated L-arabitol producing yeasts from seawater and soil samples; the yeast *P. manshurica* from seawater produced 24.6 g/L L-arabitol, while the two soil yeast isolates produced 22.5 g/L with yields of 0.615 and 0.563 g/g, respectively. Watanabe et al. [45] reported that the strain of *Candida* sp. NY7122 produced 10.69 g/L L-arabitol from 20 g/L L-arabinose when incubated at 37°C after 72 hours with a yield and productivity of 0.537 g/g and 0.148 g/L/h, respectively.

The results obtained for ethanol and L-arabitol production for the adapted *M. caribbica* Mu 2.2f were compared to similar yeasts in Tables 8 and 9. In this study, *M. caribbica* Mu 2.2f produced an ethanol concentration of 5.7 g/L with a yield and productivity of 0.338 g/g and 0.158 g/L/h, respectively, in the presence of acetic acid after 36 hours. *Ambrosiozyma monospora* produced 4.1 g/L ethanol with a yield and productivity of 0.150 g/g and 0.014 g/L/h, respectively, in the absence of acetic acid after 14 days (Table 8). McMillan and Boynton [57] reported that *C. tropicalis* NRRL Y-11860 was able to produce 8.4 g/L L-arabitol from L-arabinose in 92 hours at a yield of 1.02 g/g. This is equal to the theoretical maximum. In the present study, the adapted strain of *M. caribbica* Mu 2.2f produced L-arabitol at a yield of 0.900 g/g after 96 hours, which is close to the maximum theoretical yield (90%) as shown in Table 9. The adapted strain of *M. caribbica* Mu 2.2f has the ability to ferment L-arabinose in a shorter period to produce ethanol and L-arabitol when compared to other L-arabinose fermenting yeasts.

The ability of yeasts to ferment pentose sugars (D-xylose or L-arabinose) found in lignocellulosic biomass in the presence of inhibitors produced during pretreatment is important for second generation bioethanol production [17]. L-arabitol is considered to be of industrial importance, as it is used as natural sweetener and in clinical applications [32, 59].

*Meyerozyma caribbica* Mu 2.2f could be considered for ethanol and L-arabitol production in the presence of acetic acid. Further studies are needed on this yeast to improve ethanol and L-arabitol production. Adaptation could be considered in shake flasks at higher temperatures to overcome the problems observed in this study.

## 6. Conclusions

L-arabinose fermentation by yeasts is poorly documented compared to D-xylose. Four adapted strains of *M. caribbica* were able to ferment L-arabinose to ethanol and L-arabitol in the presence of 3 g/L acetic acid at 35°C. Adaptation improved the production of ethanol from L-arabinose by *M. caribbica* strains. The adapted *M. caribbica* Mu 2.2f strain produced 5.7 g/L ethanol with a yield of 0.338 g/g from L-arabinose at a K_L_a of 3.3 h^−1^. More L-arabitol than ethanol was produced at a K_L_a of 4.9 h^−1^ with a high yield of 0.900 g/g. The adapted *M. caribbica* Mu 2.2f strain could be a potential candidate in the fermentation of pentose rich lignocellulosic biomass, such as sugarcane bagasse, wheat straw, wheat bran, corn fibre, or brewery's spent grain. Therefore, the adapted *M. caribbica* Mu 2.2f strain could prove to be useful for both bioethanol and L-arabitol production under stressed conditions as compared to the documented pentose-fermenting yeasts under normal conditions.

## Figures and Tables

**Table 1:** Identification of selected pentose-fermenting yeast isolates.

Source	Species name	Isolate	Accession number	Similarity (%) ITS	Similarity (%) D1/D2
1	*Candida tropicalis*	Kp34ey	MH626009	99	99

2	*Cryptococcus terrestris*	C11Y	MH606241	99	100
		C12Y	MH605570	99	100
		CW1	MH606235	99	100
		CW2	MH606220	100	100

3	*Meyerozyma caribbica*	D4WPO1	MH607123	100	98

4		D14W2	MH606144	100	100
		D14YE1	MH607117	100	98
		D14YE2	MH607121	100	99
		D14YE6	MH608311	100	99
		D28L3	MH605998	100	98
		D28L4	MH606146	100	98
		Mu 2.2f	MH625960	99	100

1: dung beetle, 2: herbal concoctions, 3: banana wastes, 4: marula wine.

**Table 2:** Ethanol production from D-xylose and L-arabinose by fourteen yeasts able to ferment both sugars.

Yeast	Maximum ethanol from L-arabinose (g/L)	Maximum ethanol from D-xylose (g/L)	Time (hours) for L-arabinose	Time (hours) for D-xylose
*C. tropicalis* Kp42ey	0.6 ± 0.03	1.0 ± 0.38	96	48
*C. terrestris* C11Y	0.6 ± 0.01	0.8 ± 0.03	72	48
*C. terrestris* C12Y	0.6 ± 0.05	0.8 ± 0.01	72	24
*C. terrestris* CW1	0.5 ± 0.00	0.9 ± 0.05	72	48
*C. terrestris* CW2	0.5 ± 0.00	0.7 ± 0.01	48	48
*M. caribbica* D4WPO1	0.5 ± 0.01	0.9 ± 0.10	96	48
*M. caribbica* D14W2	1.9 ± 0.12	1.2 ± 0.20	72	48
*M. caribbica* D14YE1	0.5 ± 0.00	0.8 ± 0.02	72	48
*M. caribbica* D14YE2	0.6 ± 0.03	0.8 ± 0.03	72	48
*M. caribbica* D14YE6	3.9 ± 0.83	0.9 ± 0.12	72	48
*M. caribbica* D28L3	0.5 ± 0.01	0.9 ± 0.13	72	24
*M. caribbica* D28L4	0.5 ± 0.01	0.9 ± 0.02	48	48
*M. caribbica* Mu 2.2f	0.7 ± 0.21	0.8 ± 0.52	72	48
*S. stipitis* NRRL Y-7124	0.5 ± 0.01	4.5 ± 0.02	72	24

**Table 3:** Effect of acetic acid and temperature on growth of selected yeasts with L-arabinose as carbon source.

Yeasts	Acetic acid (g/L)^*a*^	Temperature (°C)^*b*^
*C. tropicalis* Kp42ey	1	35
*C. terrestris* C11Y	–	30
*C. terrestris* C12Y	–	30
*C. terrestris* CW1	–	30
*C. terrestris* CW2	–	37
*M. caribbica* D4WPO1	3	30
*M. caribbica* D14W2	3	40
*M. caribbica* D14YE1	3	37
*M. caribbica* D14YE2	3	37
*M. caribbica* D14YE6	3	40
*M. caribbica* D28L3	3	40
*M. caribbica* D28L4	3	40
*M. caribbica* Mu 2.2f	3	40
*S. stipitis* NRRL Y-7124	1	35

*a*: maximum acetic acid concentration at which the yeast was able to grow; *b*: maximum temperature where growth still occurred.

**Table 4:** Fermentation of L-arabinose by the adapted strains of *M. caribbica* in the presence of 3 g/L acetic acid at 35°C.

Strain number	Maximum ethanol (g/L)	Ethanol yield (g/g)	Maximum arabitol (g/L)	Arabitol yield (g/g)	Ethanol productivity (g/L/h)
Mu 2.2f	4.3 ± 0.60^*a*^	0.160	20.4 ± 3.1^*d*^	0.680	0.180
D28L3	0.8 ± 0.01^*b*^	0.030	22.2 ± 3.07^*c*^	0.783	0.022
D14YE1	1.2 ± 0.86^*a*^	0.050	21.6 ± 0.44^*c*^	0.764	0.050
D14YE6	0.8 ± 0.18^*a*^	0.030	22 ± 1.37^*d*^	0.733	0.033

Ethanol and L-arabitol yields are reported at maximum ethanol or L-arabitol concentrations. *a*: after 24 hours of fermentation; *b*: after 48 hours of fermentation; *c*: after 72 hours of fermentation; *d*: after 96 hours of fermentation.

**Table 5:** Fermentation of L-arabinose by the adapted strains of *M. caribbica* in the presence of 3 g/L acetic acid at 37°C.

Stain number	Maximum ethanol (g/L)	Ethanol yield (g/g)	Maximum arabitol (g/L)	Arabitol yield (g/g)	Ethanol productivity (g/L/h)
Mu 2.2f	1.7 ± 0.12^*b*^	0.221	2.2 ± 0.4^*b*^	0.117	0.047
D28L3	0.6 ± 0.01^*b*^	0.040	19.2 ± 0.11^*d*^	0.792	0.017
D14YE1	0.7 ± 0.01^*a*^	0.060	6.6 ± 0.71^*c*^	0.299	0.029
D14YE6	0.8 ± 0.18^*a*^	0.046	2.2 ± 0.008^*d*^	0.088	0.033

Ethanol and L-arabitol yields are reported at maximum ethanol or L-arabitol concentrations. *a*: after 24 hours of fermentation; *b*: after 48 hours of fermentation; *c*: after 72 hours of fermentation; *d*: after 96 hours of fermentation.

**Table 6:** Fermentation parameters of the adapted and parental yeast strains from *M. caribbica* Mu 2.2f with D-xylose, L-arabinose, or a mixture of the pentose sugars at 35°C with or without acetic acid.

Fermentation parameters	Absence of acetic acid						3 g/L acetic acid	
	Adapted			Parental			Adapted	
	D-xylose	L-arabinose	Mixture	D-xylose	L-arabinose	Mixture	D-xylose	L-arabinose
Maximum ethanol (g/L)	1.7 ± 0.5^*a*^	3.0 ± 0.4^*c*^	1.9 ± 0.2^*b*^	1.5 ± 0.7^*a*^	1.0 ± 0.11^*a*^	1.8 ± 0.5^*b*^	0.8 ± 0.1^*b*^	3.6 ± 0.1^*b*^
Ethanol yield (g/g)	0.042	0.148	0.059	0.044	0.076	0.052	0.040	0.181
Maximum arabitol (g/L)	—	16.8 ± 1.4^*d*^	4.7 ± 3.1^*d*^	—	7.4 ± 0.14^*c*^	2.2 ± 0.1^*b*^	—	20.2 ± 1.5^*e*^
Xylitol (g/L)	2.8 ± 0.4^*b*^	—	4.6 ± 1.6^*e*^	8.5 ± 0.4^*b*^	—	6.5 ± 1.3^*d*^	3.8 ± 0.7^*b*^	—
Arabitol yield (g/g)	—	0.494	0.347	—	0.325	0.381	—	0.657
Xylitol yield (g/g)	0.06	—	0.084	0.20	—	0.150	0.172	—
Ethanol productivity (g/L/h)	0.071	0.062	0.053	0.063	0.043	0.040	0.020	0.100

Ethanol and L-arabitol yields are reported at maximum ethanol or L-arabitol concentrations. *a*: after 24 hours of fermentation; *b*: after 36 hours of fermentation; *c*: after 48 hours of fermentation; *d*: after 72 hours of fermentation; *e*: after 96 hours of fermentation.

**Table 7:** Fermentation of L-arabinose by the adapted strain of *M. caribbica* Mu 2.2f at different K_L_a values with the addition of 3 g/L acetic acid at 35°C.

*K*_*L*_ *a* (h^−1^)	Maximum ethanol (g/L)	Ethanol yield (g/g)	Highest arabitol (g/L)	Arabitol yield (g/g)	Ethanol productivity (g/L/h)
2.3	3.8 ± 0.1^*b*^	0.270	9.9 ± 0.8^*c*^	0.370	0.106
3.3	5.7 ± 0.5^*b*^	0.338	18.3 ± 1.1^*c*^	0.660	0.158
4.9	4.2 ± 0.1^*a*^	0.321	26.7 ± 2.3^*c*^	0.900	0.175

The ethanol and L-arabitol yields are reported at maximum ethanol or L-arabitol concentrations. *a*: after 24 hours of fermentation; *b*: after 36 hours of fermentation; *c*: after 120 hours of fermentation.

**Table 8:** Comparison of ethanol production by the adapted *M. caribbica* Mu 2.2f with other reported L-arabinose fermenting yeasts.

Species	Yeast strain	*Y*_*p*/*s*_^et^ (g/g)^*a*^	Qp^et^ (g/L/h)^*b*^	Maximum ethanol (g/L)	Time (hours or days)^*c*^	References
*M. caribbica*	Mu 2.2f	0.338	0.660	5.7	36 hrs	This study
*M. caribbica*	D14YE6	0.120	0.051	3.7	72 hrs	This study
*Debaryomyces nepalensis*	*NCYC 3413*	0.03	0.020	2.43	120 hrs	[54]
*Candida* sp.	NY7122	0.040	0.031	0.75	72 hrs	[45]
*Ambrosiozyma monospora*	NRRL Y-148	0.150	0.014	4.1	12 days	[33]
*M. caribbica*	5XY2	0.011	0.005	0.6	120 hrs	[35]

^a^Ethanol yield, *Y*_*p*/*s*_^et^ (g/g): the relationship between ethanol (Δ*P*_ethanol_) formed from consumed L-arabinose (Δ*S*_arabinose_). ^*b*^Ethanol productivity, Qp^et^ (g/L/h): correlation between ethanol titre (g/L) and fermentation time (h). ^*c*^Fermentation time at which maximum ethanol (g/L) was produced towards the end or at the end of the fermentation process.

**Table 9:** Comparison of L-arabitol production by adapted *M. caribbica* Mu 2.2f with previously reported L-arabitol producing yeasts.

Yeast	Yeast strain	*Y*_*p*/*s*_^ara^ (g/g)^*a*^	Qp^ara^ (g/L/h)^*b*^	Maximum arabitol (g/L)	Time (hours)^*c*^	References
*M. caribbica*	Mu 2.2f	0.900	0.175	26.7	96	This study
*M. caribbica*	D14YE6	0.230	0.096	6.9	72	This study
*Candida tropicalis*	NRRL Y-11860	1.02	0.091	8.4	92	[57]
*M. caribbica*	5XY2	0.010	0.002	0.37	120	[35]
*Candida sp.*	NY7122	0.537	0.148	10.69	72	[45]
*C. arabinofermentans*	PYCC 5603^T^	0.600	0.200	58	270	[58]

^a^L-arabitol yield, *Y*_*p*/*s*_^ara^ (g/g): the relationship between ethanol (Δ*P*_arabitol_) formed from consumed L-arabinose (Δ*S*_arabinose_). ^*b*^L-arabitol productivity, Qp^ara^ (g/L/h): correlation between L-arabitol titre (g/L) and fermentation time (h). ^*c*^Fermentation time at which maximum L-arabitol (g/L) was produced towards the end or at the end of the fermentation process.

## Data Availability

The datasets used to support this study are included within the article and are available from the corresponding author upon request.
